# Prevalence of poor psychiatric status and sleep quality among frontline healthcare workers during and after the COVID-19 outbreak: a longitudinal study

**DOI:** 10.1038/s41398-020-01190-w

**Published:** 2021-04-15

**Authors:** Yifang Zhou, Hailong Ding, Yifan Zhang, Baoyan Zhang, Yingrui Guo, Teris Cheung, Brian J. Hall, Tieying Shi, Yu-Tao Xiang, Yanqing Tang

**Affiliations:** 1 Department of Psychiatry, The First Affiliated Hospital of China Medical University, Shenyang, Liaoning Province China; 2 Department of Geriatric Medicine, The First Affiliated Hospital of China Medical University, Shenyang, Liaoning Province China; 3 School of Public Health, China Medical University, Shenyang, Liaoning Province China; 4 School of Nursing, Hong Kong Polytechnic University, Hong Kong SAR, China; 5 New York University (Shanghai), Shanghai, China; 6 School of Global Public Health, New York University, New York, NY USA; 7 Department of Nursing, The First Affiliated Hospital of Dalian Medical University, Dalian, Liaoning China; 8 Unit of Psychiatry, Department of Public Health and Medicinal Administration, & Institute of Translational Medicine, Faculty of Health Sciences, University of Macau, Macao SAR, China; 9 Centre for Cognitive and Brain Sciences, University of Macau, Macao SAR, China; 10 Institute of Advanced Studies in Humanities and Social Sciences, University of Macau, Macao SAR, China

**Keywords:** Scientific community, Depression

## Abstract

Poor psychiatric status and sleep quality were common among frontline healthcare workers (FHWs) during the outbreak of the 2019 novel coronavirus disease (COVID-19), but the change in these mental health outcomes overtime remained unknown. This study compared the psychiatric status and sleep quality of FHWs during and after the COVID-19 outbreak in China. FHWs who volunteered to work in Hubei province (the COVID-19 epicenter) were assessed at baseline during the COVID-19 outbreak and re-assessed when they returned to their place of origin (Liaoning province) after the COVID-19 outbreak. Participants’ psychiatric status and sleep quality were measured with the Symptom CheckList-90 (SCL-90) and the Pittsburgh Sleep Quality Index (PSQI), respectively. A total of 494 FHWs was assessed at baseline and 462 at follow-up assessments. The prevalence of poor psychiatric status was 10.5% at baseline and increased to 14.9% at the follow-up assessment (*P* = 0.04). The corresponding figures of poor sleep quality at baseline and follow-up assessment were 16.4% and 27.9%, respectively (*P* < 0.001). Multiple logistic regression analysis found that severe fatigue (*p* = 0.003, OR = 1.266, 95% CI = 1.081–1.483), poor sleep quality (*p* < 0.001, OR = 1.283, 95% CI = 1.171–1.405), and history of pre-existing psychiatric disorders (*p* < 0.001, OR = 5.085, 95% CI = 2.144–12.06) were independently associated with higher odds of poor psychiatric status among the FHWs. Poor psychiatric status and sleep quality were common among FHWs during the COVID-19 outbreak, and the prevalence increased following their volunteer experiences. This suggests a critical need for longer-term psychological support for this subpopulation.

## Introduction

The 2019 novel coronavirus disease (COVID-19) was first reported in late 2019 in Wuhan, Hubei Province, China^1^, and was declared by the World Health Organization as a pandemic in March 2020^2^. As of 6 November 2020, there have been 86,184 confirmed COVID-19 cases in China, mostly originated in Hubei Province (the epicenter)^3^. To relieve the shortage of health professionals and control the disease transmission, over 42,600 healthcare workers were sourced from all parts of China and volunteered to combat the COVID-19 outbreak in Hubei Province^4,5^. The overwhelming clinical workload, fear of contagion, utmost concern about disease transmission, and perceived loneliness in isolation wards were associated with psychiatric problems among frontline healthcare workers (FHWs), particularly in the early stage of the COVID-19 outbreak^6–8^. An online survey found that the prevalence of depression, anxiety, insomnia, and distress symptoms was 50.7%, 44.7%, 36.1%, and 73.4%, respectively, among FHWs in China^9^. Commonly reported correlates of psychiatric problems included age, gender, having organic diseases, and pre-existing depression or anxiety^10–14^.

To lower the risk of psychiatric problems among FHWs, health authorities, psychiatric hospitals, and academic institutions and societies in China rapidly developed a range of responses including treatment guidelines and expert consensus^15,16^, online education^17^, 24 h hotline services, online counseling, on-site crisis psychological interventions, and relevant research^18^. In addition, financial subsidy and social support were provided to FHWs, along with ensuring their safe working environment. Consequently, the prevalence of psychiatric problems among FHWs gradually reduced in subsequent studies. For instance, a survey conducted in the late stage of the COVID-19 outbreak found that the prevalence of poor mental health status, significant stress, and poor sleep quality was 10.8%, 23.2%, and 18.9%, respectively, among FHWs in Hubei province^19^.

Previous infectious disease epidemics, such as Severe Acute Respiratory Syndrome (SARS) and Ebola, were associated with increased risk of psychiatric problems during the outbreak^20–23^ and persistent psychiatric comorbidities among the FHWs following the outbreak. For example, symptoms of depression, anxiety, burnout, psychological distress, and posttraumatic stress were still common among FHWS within 1–3 years after the SARS outbreak^20,24,25^.

After the large-scale COVID-19 outbreak had been well controlled in April 2020, several small-scale COVID-19 outbreaks occurred in some areas in China, such as Heilongjiang, Beijing, Qingdao, and Xinjiang, caused by imported cases from overseas. As the COVID-19 epidemic has been well controlled in China, most FHWs who volunteered to combat the COVID-19 outbreak in Hubei province have returned to their hometowns. To date, no longitudinal studies have examined the change of FHW’s mental health status in China, which gave us the impetus to compare the mental health status of FHWs during and after COVID-19 outbreak. It is of paramount importance to develop effective preventive measures and offer timely treatment to reduce the long-term negative outcomes brought on by COVID-19. Findings emerging from this longitudinal study can be used to advise public health policy-makers how to best distribute limited mental health resources effectively.

## Participants and methods

### Study design and participants

This study was conducted between 21 February and 6 March 2020 (baseline assessment) during the COVID-19 outbreak in Hubei Province. Consecutive sampling was used. Due to the risk of transmission, traditional face-to-face interviews were not conducted; therefore, online surveys have been widely adopted^6,9,26–29^. In this study, a smartphone-based Wechat (a social media platform used by more than one billion people in China) Mini Program named PROP psychological CT system was used to collect the data. After scanning a Quick Response code, participants could complete the assessment using their smartphones. Inclusion criteria were as follows: (1) adults aged 18 years or above; (2) FHWs who had worked in the emergency medical assistance team from Liaoning to Hubei Province, to care for confirmed and suspected cases of COVID-19; and (3) participants who were able to understand the content of the assessment and provide written informed consent. All participants were re-assessed between 22 March and 14 April 2020 (follow-up assessment) using the same battery of instruments when they returned to their hometowns in Liaoning Province and during their mandatory 2-week centralized quarantine. The study protocol was approved by the Institutional Review Board of the First Affiliated Hospital of China Medical University.

### Measurements and data collection

A data collection form was used to collect participants’ basic socio-demographic and clinical characteristics, such as gender, age, educational level, marital status, parental status (“yes/no”), history of pre-existing psychiatric disorders (“yes/no”), occupation, work experience, working status, and good family support (“yes/no”). Participants’ working status was assessed by the following standardized “yes/no” questions as follows: (1) “Have you ever directly cared for COVID-19 patients?”; and (2) “Are you familiar with the crisis response protocols and relevant knowledge?”.

Psychiatric status was evaluated by the validated Chinese version of the Symptom CheckList-90 (SCL-90), which is a self-administered scale^30,31^ consisting of 90 items in 9 domains: somatization, obsessive–compulsiveness, interpersonal sensitivity, depression, anxiety, hostility, phobic anxiety, paranoid ideation, and psychoticism. Each item scored from 1 to 5 and the total score ranges from 90 to 450. Higher scores indicated poorer mental health status. A SCL-90 total score of ≥160 was considered as having “poor psychiatric status”^32^. Fatigue was measured using the numeric rating scale (NRS), ranging from “0” (no suffering from fatigue) to “10” (unbearably suffering from fatigue)^33^. A higher score indicates more severe fatigue and the score of ≥4 was considered “clinically relevant fatigue” (“having fatigue” hereafter)^34^. Sleep quality was evaluated by the Pittsburgh Sleep Quality Index (PSQI)—Chinese version^35^. The PSQI total score ranges from 0 to 21, with the score of >7 considered as “having poor sleep quality”^36^. The Cronbach’s *α* was above 0.69 for SCL-90 subscores^37^, 0.92 for the NRS on fatigue^38^, and 0.84 for PSQI^36^.

### Data analysis

The SPSS program Version 22.0 was used to analyze data. Normal distribution of continuous variables was evaluated using the Kolmogorow–Smirnov test. Basic demographic and clinical characteristics of FHWs between baseline and follow-up assessments, and between good and poor psychiatric status at follow-up were compared using *χ*^2^-tests for categorical variables, two-independent samples *t*-tests for normally distributed continuous variables, and Mann–Whitney *U*-tests for skewed continuous variables. The independent associations between poor mental health and demographic and clinical characteristics at follow-up were examined using multiple logistic regression analysis with the “enter” method. Socio-demographic and clinical variables with significant group difference in univariate analysis were entered as independent variables. Significant level was set as *p* < 0.05 for all tests (two-sided).

## Results

Of the 2054 FHWs who volunteered in Hubei Province from Liaoning Province, 521 were consecutively invited to participate in this study at baseline and 494 completed the assessment, yielding a participation rate of 94.8% (494/521). Of them, 462 completed the follow-up assessment. Supplementary Table 1 showed the comparisons of participants’ demographic and clinical characteristics between baseline and follow-up assessments. Basic demographic and clinical characteristics by psychiatric status at follow-up assessment are shown in Table 1. Kolmogorow–Smirnov tests revealed that PSQI and fatigue total scores were skewed.

**Table 1:** Basic demographic and clinical characteristics of participants who completed the follow-up assessment.

Variables	Good psychiatric status		Poor psychiatric status		Statistics		
	(*n* = 393)		(*n* = 69)				
	*n*	%	*n*	%	*χ*^2^	df	*P*
Female	314	79.9	60	87.0	1.896	1	0.168
High education (university and above)	385	98.0	69	100	1.429	1	0.487
Married	295	75.1	51	73.9	0.001	1	0.969
Having children	244	62.1	48	69.6	1.412	1	0.235
Nurse	292	74.3	52	75.4	0.035	1	0.852
Working more than 5 years	328	83.5	62	89.9	1.824	1	0.177
Working in Hubei ≥ 8 weeks	193	49.1	30	43.5	0.745	1	0.388
Caring for critical COVID-19 patients	143	36.4	48	40.6	1.231	1	0.267
Familiar with crisis response knowledge					6.638	2	**0.036**
Very familiar	236	60.1	52	75.4			
Familiar	121	30.8	15	21.7			
Not familiar	36	9.2	2	2.9			
Good family support	357	90.8	55	10.2	7.533	1	**0.006**
History of pre-existing psychiatric disorders	16	4.1	16	23.2	33.275	1	**<0.001**
	Mean	SD	Mean	SD	*T*/*Z*	df	*P*
Age (years)	35.14	7.044	36.55	7.101	1.535	460	0.125
Fatigue total score	3.04	2.268	4.88	1.867	−8.188	--- ^a^	**<0.001**
PSQI total score	4.85	3.028	8.72	3.635	−6.121	--- ^a^	**<0.001**

*df* degrees of freedom, *PSQI* Pittsburgh Sleep Quality Index.

^a^Mann–Whitney *U*-tests. Bolded values < 0.05.

Overall psychiatric status with the domain scores and poor sleep quality of participates were compared between baseline and follow-up assessments (Table 2). Compared with the baseline assessment, the prevalence of poor mental health significantly increased at follow-up assessment [10.5% (95% confidence interval (95% CI) 7.8–13.2%) at baseline vs. 14.9% (95% CI 11.7–18.2%) at follow-up; *p* = 0.04]. The prevalence of poor sleep quality also significantly increased [16.4% (95% CI 13.1–19.8%) at baseline vs. 27.9% (95% CI 23.8–32%) at follow-up; *p* < 0.001].

**Table 2:** Comparisons of the SCL-90 total and domain scores between the assessments in Hubei and Liaoning Provinces.

	Baseline (*n* = 494)	Follow-up (*n* = 462)	Statistics		
	*N* (%)	*N* (%)	*χ*^2^	df	*P*
SCL-90 total score (≥160)	52 (10.5)	69 (14.9)	4.197	1	**0.04**
Somatization	46 (9.3)	52 (11.3)	0.98	1	0.322
Obsession	90 (18.2)	101 (21.9)	1.982	1	0.159
Interpersonal relation	44 (8.9)	56 (12.1)	2.634	1	0.105
Depressed	44 (8.9)	58 (12.6)	3.332	1	0.068
Anxious	37 (7.5)	43 (9.3)	1.028	1	0.311
Hostility	32 (6.5)	35 (7.6)	0.442	1	0.506
Terrifying	86 (17.4)	93 (20.1)	0.297	1	0.586
Bigoted	18 (3.6)	36 (7.8)	7.709	1	**0.005**
Mental degeneration	20 (4)	27 (5.8)	1.647	1	0.199
Additional items (diet/sleep)	86 (17.4)	93 (20.1)	1.161	1	0.281
PSQI total score (>7)	77 (16.4)	129 (27.9)	17.876	1	**<0.001**

*df* degrees of freedom, *PSQI* Pittsburgh Sleep Quality Index, *SCL-90* Symptom CheckList-90.

Univariate analyses revealed that there were significant differences between good and poor psychiatric status in terms of knowledge about crisis response, family support, history of pre-existing psychiatric disorders, fatigue, and PSQI total score (Table 1). Poorer sleep quality (odds ratio (OR) = 1.350, 95% CI = 1.211–1.506, *P* < 0.001) and history of psychiatric disorders (OR = 3.384, 95% CI = 1.218–9.401, *P* = 0.019) were independently and positively associated with poor psychiatric status at baseline (Supplementary Table 2). Multiple logistic regression analysis revealed that participants who reported more severe fatigue (OR = 1.266, 95% CI = 1.081–1.483, *P* = 0.003), poorer sleep quality (OR = 1.283, 95% CI = 1.171–1.405, *P* < 0.001), and history of psychiatric disorders (OR = 5.085, 95% CI = 2.144–12.06, *P* < 0.001) were independently and positively associated with poor psychiatric status at follow-up (Table 3).

**Table 3:** Factors independently associated with poor mental health at follow-up.

Variables	Multivariate regression analysis		
	*P*-value	OR	95% CI
Fatigue total score	**0.003**	1.266	1.081–1.483
PSQI total score	**<0.001**	1.283	1.171–1.405
Crisis response knowledge			
Not familiar	–	–	1.0
Familiar	0.226	2.797	0.529–14.777
Very familiar	0.059	4.616	0.941–22.645
Good family support	0.054	0.426	0.178–1.016
History of pre-existing psychiatric disorders	**<0.001**	5.085	2.144–12.06

*CI* confidential interval, *OR* odds ratio, *PSQI* Pittsburgh Sleep Quality Index.

Bolded values < 0.05.

## Discussion

To the best of our knowledge, this was the first longitudinal study that assessed psychiatric status and sleep quality of FHWs during and after the COVID-19 outbreak. Our results showed that the prevalence of poor psychiatric status and sleep quality among FHWs significantly increased after the COVID-19 outbreak. The psychiatric status was evaluated with the SCL-90, as this is a comprehensive instrument on general mental health in nine domains and has been widely used in clinical services and research^11,39–41^, with satisfactory psychometric properties in Chinese populations^37^.

The increased prevalence of poor psychiatric status and sleep quality among FHWs after the COVID-19 outbreak could be attributed to several reasons. First, FHWs may have delayed psychiatric reactions to overwhelming clinical workload. For instance, coupled with excessive fear of contagion, posttraumatic stress disorder (PTSD) may occur among FHWs after the COVID-19 outbreak^24^. PTSD patients with delayed onset usually develop full symptoms weeks and months after the event^42^. In this study, we speculated that once the FHWs finished their heavy clinical duty, their psychological defense mechanism may relax, allowing them to recall the traumatic images of their clinical work, which could worsen their psychiatric status. Second, when FHWs worked in Hubei Province, they focused on their clinical duties without any interference of other psychological pressure. However, once they returned to their original working environments, they resumed facing great family pressure, dealing with complex interpersonal/collegial relationships, and academic competition. All these stressors could elevate the risk of psychiatric problems. This assumption was affirmed in a previous study that showed around 30% of clinicians developed new-onset episodes of psychiatric disorders, such as depression, anxiety, and substance use, after the SARS outbreak^43^. Another possible reason for exacerbation of symptoms could be their experience of being quarantined, which is associated with increased psychiatric symptoms^44^.

In this study, poor sleep quality was positively associated with poor psychiatric status, which is consistent with previous findings^45–47^. Poor sleep quality was not only common in patients with psychiatric disorders, but was considered as a predictor of future occurrence of certain psychiatric disorders, such as depression and anxiety^48,49^. Some researchers suggested that early recognition and treatment of poor sleep quality may lower the risk of future psychiatric disorders^50^. As expected, both fatigue and history of psychiatric disorders were significantly associated with higher risk of poor psychiatric status. Fatigue is a common symptom of certain psychiatric disorders, such as anxiety and depression^42^, and was significantly associated with lower quality of life^51^. Appropriate psychosocial interventions, such as cognitive behavioral therapy^52^, could improve fatigue. High stress and related problems of FHWs with a history of psychiatric disorders may trigger their pre-existing disorders or worsen residual psychiatric symptoms, which is supported by previous findings^27,53^.

The strengths of this study included the longitudinal design and use of validated and widely used assessment tools on psychiatric status and sleep disturbance. However, some limitations should be acknowledged. First, only one follow-up assessment was conducted in this study and hence, the long-term impact of COVID-19 outbreak on FHWs’ psychiatric status and sleep quality could not be ascertained. Second, due to logistical reasons, only standardized scales were administered to evaluate FHW’s psychiatric status, rather than using diagnostic instruments. In addition, some factors related to psychiatric status of FHWs, such as the length of stay in Hubei at the baseline assessment, were not recorded. Finally, FHWs in only one province (Liaoning Province) was recruited in this study, which may limit the generalizability of the findings to other regions of China.

In conclusion, poor psychiatric status and sleep quality were common among FHWs during the COVID-19 outbreak, and the prevalence of these conditions may increase after the COVID-19 outbreak. Long-term sustainable psychological support should be provided for this subpopulation. Prospective studies with longer follow-up period should be conducted to examine the long-term impact of the COVID-19 outbreak on FHWs’ psychiatric status and sleep quality.

## Supplementary information

SUPPLEMENTAL MATERIAL

## Supplementary information

**Supplementary Information** The online version contains supplementary material available at (10.1038/s41398-020-01190-w).
