# Supplementary material for: Prevalence of poor psychiatric status and sleep quality among frontline healthcare workers during and after the COVID-19 outbreak: a longitudinal study
Source: Transl Psychiatry. 2021 Apr 15;11:223. doi: 10.1038/s41398-020-01190-w (PMC8047599; doi:10.1038/s41398-020-01190-w)
Supplement: Supplementary file 1 — SUPPLEMENTAL MATERIAL [file 41398_2020_1190_MOESM1_ESM.pdf]

Supplementary Table 1. Demographic and clinical characteristics between FHWs working in Hubei during the COVID-19 outbreak and in Liaoning after the COVID-19.

Variables	Baseline (N=494)		Follow-up (N=462)		Statistics		
	n	%	n	%	X^2	df	P
Female	410	83	374	81	0.676	1	0.411
High education (university and above)	481	97.4	454	98.3	0.9	1	0.343
Married	323	66.3	324	71.2	2.609	1	0.106
Having children	282	57.1	292	63.2	13.725	1	0.054
Nurse	415	84	344	74.5	13.306	1	**<0.001**
Working more than 5 years	402	81.4	390	84.4	1.551	1	0.213
Working in Hubei ≥ 8 weeks	---	---	223	48.3	0.745	1	0.388
Caring for critical COVID-19 patients	221	44.7	171	37	5.887	1	**0.015**
Familiar with crisis response knowledge					35.631	2	**<0.001**
Very familiar	389	78.7	288	62.3			
Familiar	93	18.8	136	29.4			
Not familiar	12	2.4	38	8.2			
Good family support	417	84.4	412	89.2	4.704	1	**0.03**
History of psychiatric disorders	24	5.7	32	6.9	0.544	1	0.461
	Mean	SD	Mean	SD	T/Z	df	P
Age (years)	33.76	6.69	35.35	7.1	-3.578	954	**<0.001**
Fatigue total score	---	---	3.31	2.3	-8.188	---	---
PSQI total score	4.77	3.05	5.93	3.6	-5.078	---	**<0.001**

PSQI Pittsburgh Sleep Quality Index. Bolded values <0.05.

Supplementary Table 2. Factors independently associated with poor mental health at baseline.

Variables	Multivariate regression analysis		
	P value	OR	95% CI
PSQI total score	**<0.001**	1.350	1.211-1.506
Crisis response knowledge			
Not familiar	-	-	1
Familiar	0.08	0.209	0.036-1.207
Very familiar	0.052	0.203	0.041-1.011
History of pre-existing psychiatric disorders	**0.019**	3.384	1.218-9.401

CI confidential interval; OR: odds ratio; PSQI Pittsburgh Sleep Quality Index.

Bolded values <0.05.
